# HBeAg Is Indispensable for Inducing Liver Sinusoidal Endothelial Cell Activation by Hepatitis B Virus

**DOI:** 10.3389/fcimb.2022.797915

**Published:** 2022-01-31

**Authors:** Xiaohong Xie, Jinzhuo Luo, Dan Zhu, Wenqing Zhou, Xuecheng Yang, Xuemei Feng, Mengji Lu, Xin Zheng, Ulf Dittmer, Dongliang Yang, Jia Liu

**Affiliations:** ^1^ Department of Infectious Diseases, Union Hospital, Tongji Medical College, Huazhong University of Science and Technology, Wuhan, China; ^2^ Institute for Virology, University Hospital of Essen, University of Duisburg-Essen, Essen, Germany

**Keywords:** hepatitis B virus (HBV), hepatitis B e antigen (HBeAg), liver sinusoidal endothelial cells (LSECs), CD8 T cell, tumor necrosis factor (TNF)

## Abstract

**Background and Aims:**

Liver sinusoidal endothelial cells (LSECs) serve as sentinel cells to detect microbial infection and actively contribute to regulating immune responses for surveillance against intrahepatic pathogens. We recently reported that hepatitis B e antigen (HBeAg) stimulation could induce LSEC maturation and abrogate LSEC-mediated T cell suppression in a TNF-α and IL27 dependent manner. However, it remains unclear how HBeAg deficiency during HBV infection influences LSEC immunoregulation function and intrahepatic HBV-specific CD8 T cell responses.

**Methods:**

The function of LSECs in regulating effector T cell response, intrahepatic HBV-specific CD8 T cell responses and HBV viremia were characterized in both HBeAg-deficient and -competent HBV hydrodynamic injection (HDI) mouse models.

**Results:**

LSECs isolated from HBeAg-deficient HBV HDI mice showed a reduced capacity to promote T cell immunity *in vitro* compared with those isolated from wild-type HBV HDI mice. HBeAg expression replenishment in HBeAg-deficient HBV HDI mice restored the HBV-induced LSEC maturation, and resulted in potent intrahepatic anti-HBV CD8 T cell responses and efficient control of HBV replication. Moreover, *in vivo* TNF-α, but not IL27 blockade in HBV HDI mice impaired HBV-specific CD8 T cell immunity and delayed HBV clearance.

**Conclusion:**

Our study underlines that HBeAg is indispensable for HBV-induced LSEC maturation to trigger intrahepatic HBV-specific T cell activation, and provides a new mechanism to elucidate the intrahepatic immune microenvironment regulation upon HBV exposure.

## Introduction

The liver induces immune tolerance rather than responses to antigens encountered locally under physiological conditions (Protzer et al., 2012). The unique immune microenvironment of the liver is largely determined by organ-resident parenchymal and non-parenchymal antigen-presenting cells (APCs), among which LSECs are one of the most important non-parenchymal APCs with the largest number (Crispe, 2011). The location of LSECs allows them to intensively interact with passenger lymphocytes and present antigens to T cells efficiently (Shetty et al., 2018). T cell priming by LSECs may induce distinct T cell differentiation programs, such as generating regulatory CD4 T cells (Carambia et al., 2014), causing CD8 T cell tolerance (Schurich et al., 2009; Hochst et al., 2012), or inducing memory-like CD8 T cells (Bottcher et al., 2013). Moreover, direct contact with LSECs can result in strong suppression of effector T cell activation (Liu et al., 2013). LSECs also act as a platform for anchoring blood-borne effector CD8 T cells through platelet-mediated docking (Guidotti et al., 2015). Thus, LSECs have been considered to play a crucial role in contributing to liver immune surveillance against infection by modulating the activation of local immune cells (Wohlleber and Knolle, 2016).

Chronic HBV infection remains a major public health issue worldwide. It has been shown that HBV uses multiple mechanisms including the inherent tolerogenic property of the liver to dampen host adaptive immunity, especially the intrahepatic anti-HBV T cell responses, to facilitates its persistence (Thomson and Knolle, 2010; Bertoletti and Ferrari, 2016; Maini and Burton, 2019). However, it remains largely unknown how the T cell regulation function of LSECs changes during HBV infection. Recently, we for the first time reported that LSECs switch their immunomodulation status upon HBV exposure. In response to HBeAg stimulation, LSECs undergo immunological maturation and become permissive to effector T cell activation. Both HBV- and HBeAg-exposed LSECs produced increased TNF-α and IL27, which abolished the inhibition of T cell IFNγ production by LSECs (Xie et al., 2021). However, it remains unclear how HBeAg deficiency during HBV infection influences LSEC immunoregulation function and intrahepatic HBV-specific CTL responses.

In the current study, we further investigated whether HBeAg is indispensable for HBV inducing LSECs maturation by using the HBV hydrodynamic injection (HDI) mouse model. We observed that HBeAg-deficient HBV failed to induce LSEC maturation and led to attenuated intrahepatic HBV-specific CD8 T cell responses. TNF-α produced by matured LSECs may play a pivotal part in triggering intrahepatic HBV-specific CD8 T cell responses and HBV clearance.

## Materials and Methods

### Mice

Male wild-type C57BL/6 mice were purchased from Hunan Slack King Laboratory Animal Co., Ltd. (Changsha, China). TLR2 knock out mice were generously provided by Professor Sha Wu (Southern Medical University, Guangzhou, China). All animals were bred and kept under specific pathogen-free (SPF) conditions in the Animal Care Center of Tongji Medical College (Wuhan, China).

### Plasmids, Antibodies, and Reagents

The antibodies and primers used in this study are listed in **Supplementary Table 1** and **Supplementary Table 2**. All plasmids were prepared using Endo-Free Plasmid Kits (Omega, Norcross, GA, USA). The levels of HBeAg and hepatitis B surface antigens (HBsAg) in the serum were determined by the corresponding ELISA kits (Kehua, Shanghai, China). HBV DNA copies were measured by a diagnostic kit for HBV DNA (Sansure, Changsha, China) using quantitative real-time PCR (qRT-PCR). Total RNA was isolated using RNAiso Plus (Takara, Shiga, Japan). One-step RT-PCR was carried out with the One Step SYBR^®^ PrimeScript™ RT- PCR Kit II (Takara) on the iCycler real-time amplification system (Bio-Rad, Hercules, CA, USA). All of the above operations were performed according to the manufacturer’s instructions.

### Hydrodynamic Injection in Mice

Hydrodynamic injection was performed as described previously by using plasmids to establish HBV replication in mice (Wang et al., 2018). In brief, male mice (6 to 8 weeks of age) were injected with 10 μg plasmids (except for pCI-neo/null and pCI-neo/HBeAg plasmid with 20 ug) in a volume of phosphate buffer saline (PBS) equivalent to 0.1 mL/g of the mouse body weight through the tail vein within 5-8 seconds.

### Mouse Cell Isolation and *In Vitro* Stimulation

Isolation of mouse LSECs was performed as described previously with a cell purity over 95% (Liu et al., 2017), and less than 0.5% of NK cells and DCs were found in the purified LSECs by using this isolation procedure (Liu et al., 2013). Isolation of splenocytes and intrahepatic infiltrated lymphocytes was performed as described previously (Yang et al., 2019). All isolated cell fractions contained less than 5% dead cells. 10 μg/ml H-2Kb-restricted HBcAg-derived CD8 epitope peptide core93-100 (MGLKFRQL), or HBsAg derived CD8 epitope peptide env208-216 (ILSPFLPLL) was added to the culture system at 37°C for 5 h *in vitro*.

### Flow Cytometry

Cell surface and intracellular staining for flow cytometry analysis was performed as described previously (Liu et al., 2018; Yang et al., 2019). The antibodies used for surface and intracellular staining are listed in **Supplementary Table 2**. Freshly isolated cells were used for all assays, and data were acquired using a FACS Canto II flow cytometer (BD Biosciences, San Jose, CA, USA) and analyzed using FlowJo software (Tree Star, Ashland, OR, USA). Cell debris and dead cells were excluded from the analysis based on scatter signals and Fixable Viability Dye eFluor 506 (eBioscience, San Jose, CA, USA).

### Cytokine Assays

LSECs were cultured 5×10^5 cells per well pre-stimulated with or without recombinant HBeAg (ProSpec, Ness-Ziona, Israel) in a total volume of 500μl, cell-free supernatants were collected and subjected to assays to measure cytokines using cytokine ELISA kits (eBioscience) or cytometric bead array (CBA) cytokine kit (BD Biosciences). Red blood cell-depleted splenocytes were cultured 1×10^6 cells per well with or without LSECs at a ratio of 2:1 (splenocytes to LSECs) in a total volume of 500μl. Splenocytes were stimulated with 1 μg/mL anti-CD3 and 1 μg/mL anti-CD28 (BD Bioscience).

### TNF-α Blockade in Mice

For TNF-α blockade, mice were intravenously injected with anti-TNF-α (200 ug per mouse, clone XT3.11, BioXcell, Lebanon, NH, USA) every two days for 10 days.

### Statistical Analysis

Statistical analyses were performed using the SPSS statistical software package (version 22.0, SPSS Inc., Chicago, IL, USA). The Shapiro-Wilk method was used to test for normality. Parametric analysis methods were used when the data were normally distributed; otherwise, non-parametric tests were employed. Unpaired t test, one-way ANOVA, were used where appropriate. All reported p values were two-sided, and a p value less than 0.05 was considered statistically significant.

## Results

### Abolishment of LSEC-Mediated T Cell Suppression by HBV Is HBeAg-Dependent

**Figure 1:**
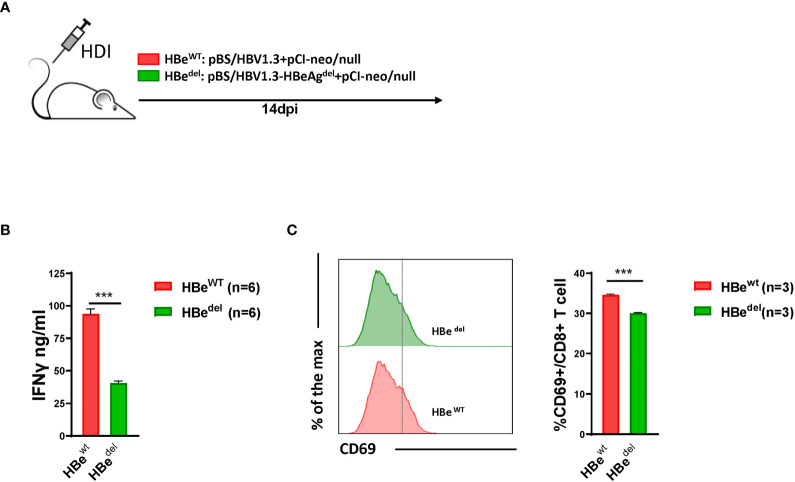
Abolishment of LSEC-mediated T cell suppression by HBV is HBeAg-dependent **(A)** Experimental scheme of the HBeAg normal expression HBV hydrodynamic injection mouse model and HBeAg expression-deficient HBV hydrodynamic injection mouse model. C57BL/6 mice are hydrodynamically injected with pBS/HBV1.3 plus pCI-neo/null (HBe^wt^) or pBS/HBV1.3-HBeAg^del^ plus pCI-neo/null (HBe^del^). **(B)** LSECs from mice hydrodynamically injected with different combinations of plasmids are separated and cocultured with polyclonal stimulated splenocytes at a ratio of 1:2 (LSECs to splenocytes). IFNγ production is measured after 48 h. **(C)** CD8 T cells in the liver are analyzed for CD69 expression by flow cytometry at 14 days post HDI (dpi). Data are representative of 3 independent experiments. Unpaired t-test is used. ***p < 0.001.

As mentioned above, our previous data showed that both *in vivo* and *in vitro* HBeAg exposure could abolish LSEC-mediated T cell suppression (Xie et al., 2021). To further characterize the role of HBeAg in inducing LSEC activation during HBV infection, we constructed an HBeAg expression deficiency plasmid (pBS/HBV1.3-HBe^del^) by engineering a G to A mutation at nucleotide 1896 of the HBV genome in plasmid pBS/HBV1.3. The G1896A mutation converts codon 28 of the pre-core sequence from UGG to the UAG stop codon, which is believed to cause HBeAg-negative HBV infection in patients (Carman et al., 1989; Gu et al., 2019). As shown in **Figure S1A**, mice receiving pBS/HBV1.3-HBe^del^ plasmid HDI (HBe^del^ mice) showed no detectable HBeAg in the serum, whereas those with pBS/HBV1.3 HDI (HBe^wt^ mice) showed sustained HBeAg expression. LSECs separated from HBe^del^ mice significantly suppressed the IFNγ production of activated T cells compared with those from HBe^wt^ HBV HDI mice (**Figure 1B**). Consistent with these findings, we observed that intrahepatic CD8 T cells of HBe^del^ mice showed significantly lower expression of the activation marker CD69 compared with those of HBe^wt^ mice (**Figure 1C**). Taken together, these results suggest that HBeAg is essential for the HBV-induced LSEC activation to exert intrahepatic anti-HBV CD8 T cell responses.

### HBeAg Deficiency Deprives HBV-Specific CTLs Effector Function

**Figure 2:**
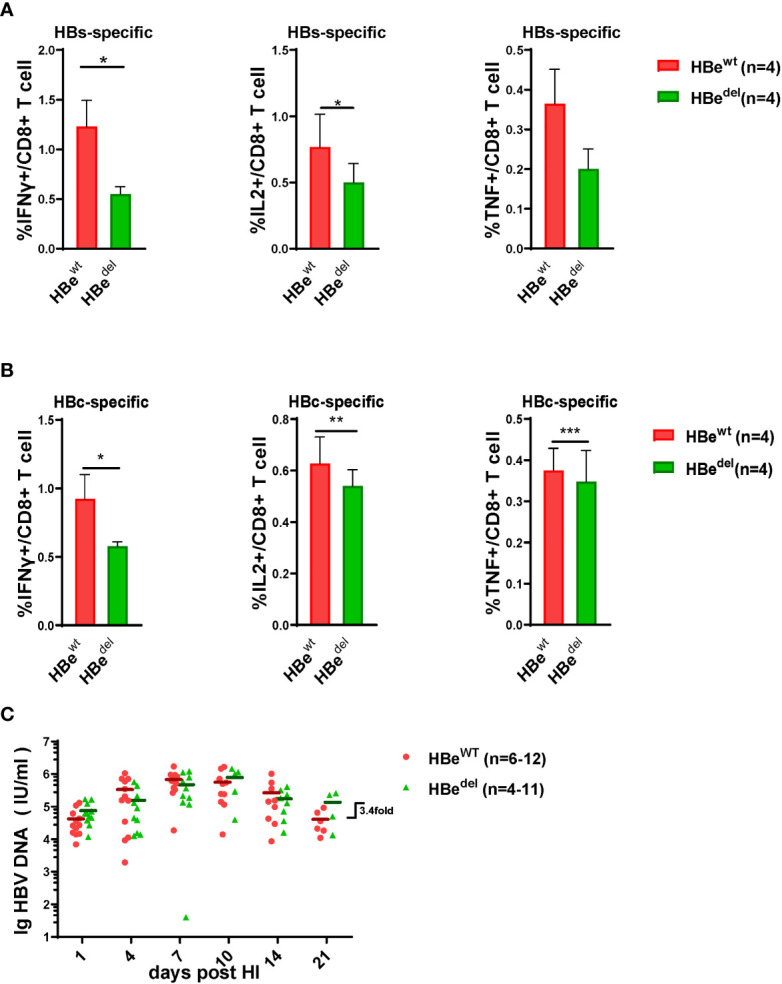
HBeAg deficiency deprives HBV specific CTLs effector function **(A, B)** Liver-infiltrating lymphocytes are separated 21 dpi and stimulated with HBsAg epitope peptide (env208-216: ILSPFLPLL) or HBcAg epitope peptide (core93-100: MGLKFRQL) for 5 h *in vitro*. Intracellular staining is performed, and the frequencies of HBs(A)/HBc(B)-specific-IFNγ+, IL2+, and TNF-α + CD8 T cells are shown. **(C)** The dynamic virus levels of the HBe^wt^ mice (n=12 at 1dpi) and the HBe^del^ mice (n=11 at 1dpi) at different time points. Data are representative of 3 independent experiments. Error bars, mean ± SEM. Unpaired t-test is used. *p < 0.05; **p < 0.01; ***p < 0.001.

The inhibition effect of HBeAg deficiency on LSECs activation has been examined, following, we determined if the effector function of HBV specific CTLs cell was suppressed in the absence of HBeAg. By intracellular flow cytometry we found that after HBsAg or HBcAg epitope peptide stimulation, LSECs separated from HBe^del^ mice induced IFNγ/IL2 and TNF-α expression in specific CTLs more decreased than those from HBe^wt^ mice (**Figures 2A, B**). At 21 days post HDI (dpi), the serum HBV DNA titers of HBe^del^ mice were 3.4-fold higher than those of HBe^wt^ mice (**Figure 2C**). These findings indicated that HBeAg is essential for the induction of LSEC activation to promote HBsAg- and HBcAg specific CTLs effector function, and accelerate serum HBV DNA clearance.

### HBeAg Replenishing Restores LSEC Activation in HBeAg-Deficient HBV HDI Mice

**Figure 3:**
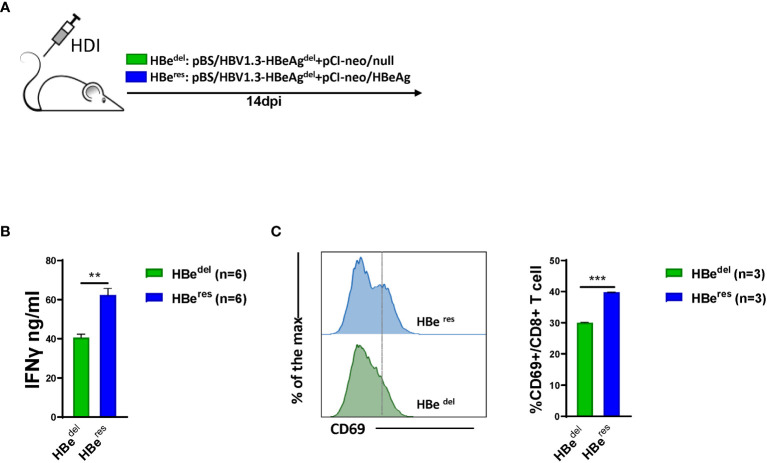
HBeAg replenishing restores LSEC activation in HBeAg-deficient HBV HDI mice **(A)** C57BL/6 mice are hydrodynamically injected with pBS/HBV1.3-HBeAg^del^ plus pCI-neo/null (HBe^del^) or pBS/HBV1.3-HBeAg^del^ plus pCI-neo/HBeAg (HBe^res^). **(B)** LSECs from mice hydrodynamically injected with different combinations of plasmids are separated at 14 dpi and cocultured with polyclonal stimulated splenocytes at a ratio of 1:2 (LSECs to splenocytes). IFNγ production is measured after 48 h. **(C)** Intrahepatic CD8 T cells are analyzed for CD69 expression by flow cytometry at 14 dpi. Data are representative of 3 independent experiments. Error bars, mean ± SEM. Unpaired t-test is used. **p < 0.01; ***p < 0.001.

**Figure 4:**
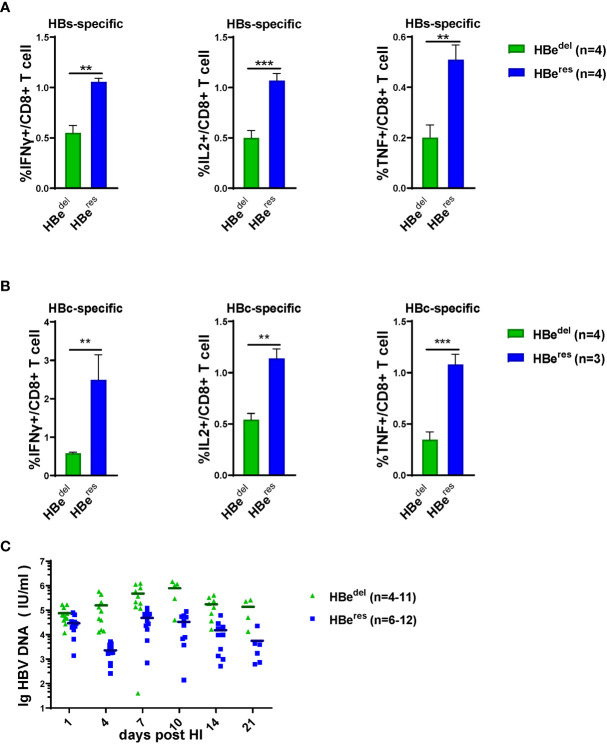
HBeAg replenishing induces potent intrahepatic anti-HBV CTLs responses Liver-infiltrating lymphocytes are separated 21 dpi and stimulated with HBsAg epitope peptide (env208) or HBcAg epitope peptide (core93) for 5 h *in vitro*. Intracellular staining is performed, and the frequencies of HBs **(A)** /HBc **(B)**-specific-IFNγ+, IL2+, and TNF-α+ CD8 T cells are shown. **(C)** The dynamic virus levels of the HBe^del^ mice (n=11 at 1dpi) and the HBe^res^ mice (n=12 at 1dpi) at different time points. Data are representative of 3 independent experiments. Error bars, mean ± SEM. Unpaired t-test is used. **p < 0.01; ***p < 0.001.

We next performed further experiments to distinguish whether LSEC reactivate to promote CD8 T cell response after HBeAg replenishing in HBeAg-deficient HBV HDI mice (**Figure 3A**). As shown in **Figure S1A**, HDI of plasmid pCI-neo/HBeAg together with pBS/HBV1.3-HBe^del^ (HBe^res^) resulted in significant HBeAg production in mice. The concentration of serum HBeAg detected in HBe^res^ mice peaked at 1 dpi and started to drop afterwards. Serum HBeAg could still be detected in HBe^res^ mice at low levels at 10 dpi and turned negative at 14 dpi. The concentration of serum HBeAg was low in HBe^wt^ mice at 1 dpi and started to gradually increase during the observation period. Similar levels of serum HBeAg between HBe^res^ mice and HBe^wt^ mice were detected at 4 dpi (**Figure S1A**). We observed that restoring HBeAg expression by HDI of plasmid pCI-neo/HBeAg could partially abolish LSEC-mediated T cell suppression in HBe^del^ mice (**Figure 3B**). Restoring HBeAg expression in HBe^del^ mice (HBe^res^ mice) resulted in increased CD69 expression in intrahepatic CD8 T cells (**Figure 3C**) and even further enhanced HBcAg- and HBsAg-specific CD8 T cell responses compared with those of HBe^del^ mice (**Figures 4A, B**). Furthermore, HBe^res^ mice induced more potent CD69 expression in spleen CD8 T cells both than HBe^del^ and HBe^wt^ mice (**Figure S1B**). Accordingly, the serum HBV DNA titers of HBe^res^ mice were significantly lower compared to those of HBe^del^ mice (**Figure 4C**). In sum, these results reinforce that HBeAg is crucial to the induction of LSEC activation during HBV infection.

### TNF-α Blockade Results in Decreased Intrahepatic HBV-Specific CD8 T Cell Responses and Delayed HBV Clearance

**Figure 5:**
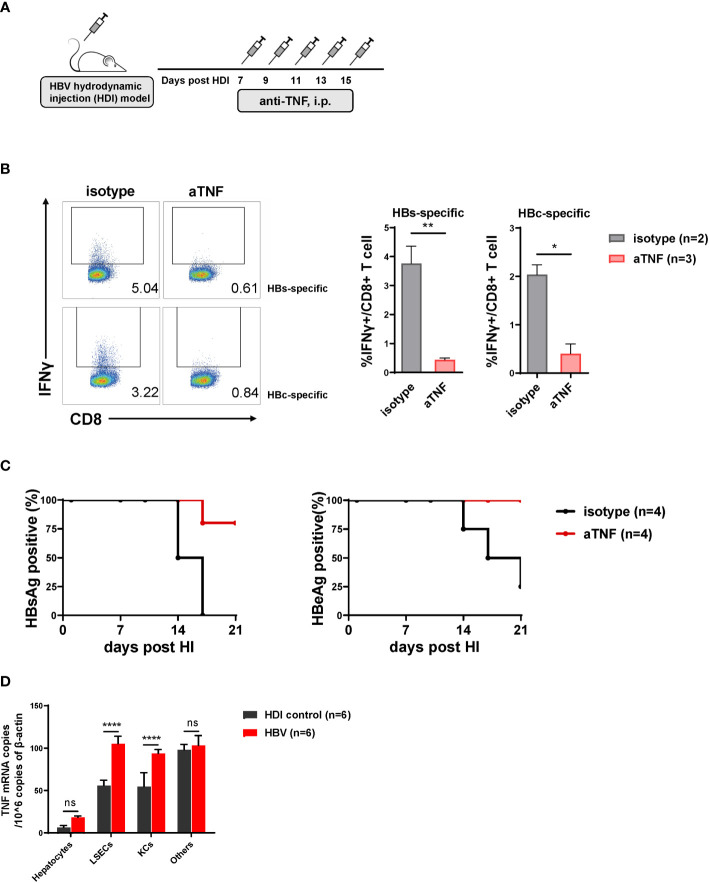
TNF-α blockade results in decreased intrahepatic hepatitis B virus (HBV)-specific CD8 T cell responses and delayed HBV clearance. **(A)** C57BL/6 mice are hydrodynamically injected with the pSM2 plasmid and intraperitoneally injected with anti-TNF-α antibody (200 ug per mouse) or isotype control antibody (control) every 2 days from 7 to 15 dpi. **(B)** Liver-infiltrating lymphocytes are separated from HBV plasmid- and anti-TNF-α-injected mice at 22 dpi and stimulated with HBsAg epitope peptide (env208) or HBcAg epitope peptide (core93) for 5 h *in vitro*. Intracellular staining is performed, and the frequencies of IFNγ+ CD8 T cells are shown. **(C)** The serum HBsAg and HBeAg levels in the plasmid-injected mice are monitored at indicated time points. Data are shown as the kinetic of HBsAg or HBeAg positive percentage at indicated time points. **(D)** Hepatocytes, LSECs, Kupffer cells (KCs), and other cells are separated from mice hydrodynamically injected with pSM2 plasmid (HBV) or PBS (HDI control) at 14 dpi, and total RNA of the cells are extracted for testing TNF-α mRNA by quantitative real-time PCR (RT-PCR). Data are representative of 3 independent experiments. Error bars, mean ± SEM. Unpaired t-test is used. *p < 0.05; **p < 0.01; ***p < 0.0001, ns, not significant (p>0.05).

We have identified that HBeAg activated LSECs produce TNF-α and IL27 to exert T cell immunity (Xie et al., 2021). To further dissect the role of TNF-α and IL27 in mediating intrahepatic anti-HBV T cell responses and HBV clearance, we blocked the function of TNF-α and IL27 *in vivo* in the course of HBV clearance by treating HBV HDI mice with corresponding blocking antibodies (**Figure 5A**). We observed that TNF-α blocking antibody treated mice showed significantly decreased percentages of IFNγ producing CD8 T cells in the liver in response to HBsAg or HBcAg epitope peptide restimulation compared with those in control mice (**Figure 5B**). Moreover, 80% and 100% TNF-α blocking antibody-treated mice remained HBsAg- and HBeAg-positive at 21 dpi, while all control mice cleared HBsAg and 80% cleared HBeAg at this time point, demonstrating that TNF-α blockade could significantly delay the serum HBsAg and HBeAg clearance in HBV HDI mice (**Figure 5C**). However, no significant differences in HBV clearance were observed between mice treated with IL27 blocking antibodies and control mice (**Figure S2A**). Collectively, these findings suggest that TNF-α, but not IL27, plays a key role in inducing intrahepatic anti-HBV CD8 T cell responses and lead to accelerating HBV clearance.

We further explored whether TNF-α was produced by LSECs or other cells during the process. Indeed, the TNF-α mRNA expression showed significantly different between HBV mice and HDI control mice in LSECs and Kupffer cells (KCs), but not hepatocytes nor other cells (**Figure 5D**). However, KCs could not trigger CD8 T cells responses when HBeAg stimulation (**Figure S3A**). Altogether, these results confirm that HBeAg induced LSECs to mediate anti-HBV immunity is partially by upregulating TNF-α expression of LSECs.

### HBeAg Induces LSEC to Trigger T Cell Immunity Is Independent of the TLR2 Signaling Pathway

**Figure 6:**
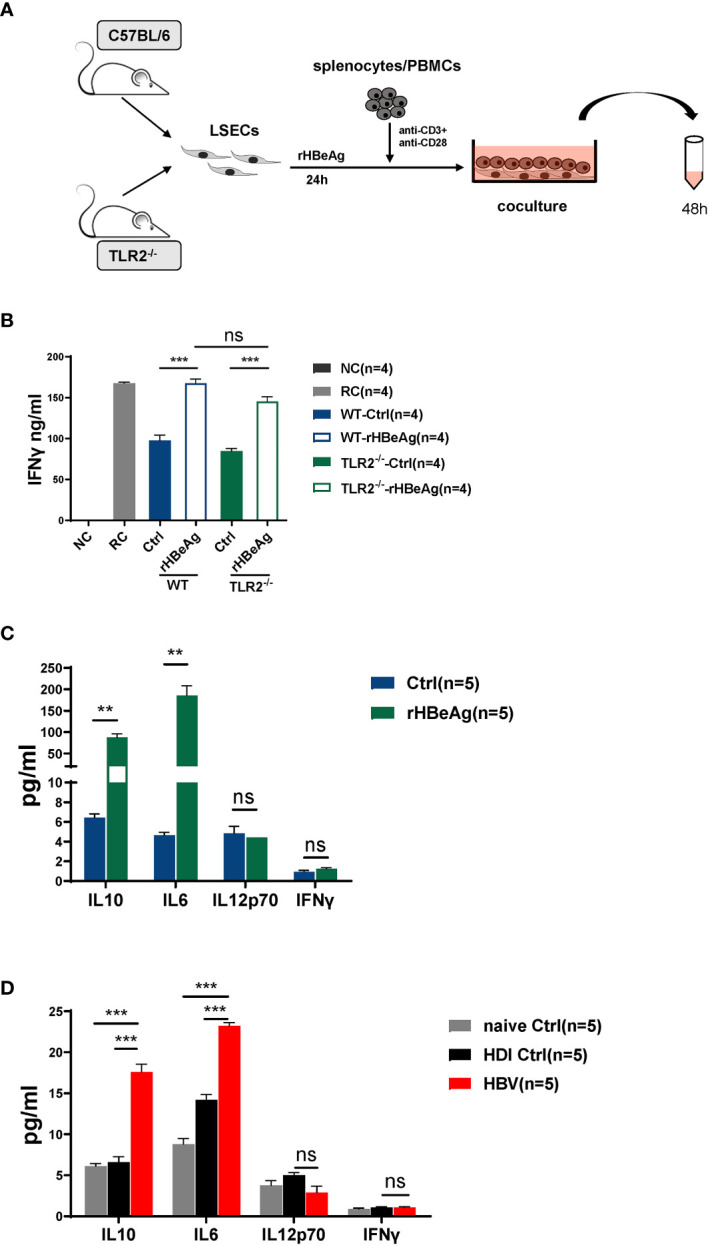
HBeAg induces LSEC to trigger T cell immunity is independent of the TLR2 signaling pathway **(A, B)** LSECs are separated from naïve C57BL/6 mice and toll like receptor 2 (TLR2) knockout mice and stimulated with 10μg/ml rHBeAg or not for 24h, then washed and cocultured with polyclonal stimulated splenocytes at a ratio of 1:2 (LSECs: splenocytes). IFNγ production is measured after 48h. Anti-CD3/anti-CD28-stimulated splenocytes only is used as responder controls (RC). Unstimulated splenocytes is used as negative control (NC). **(C)** LSECs from naïve mice are pretreated with 10 μg/ml rHBeAg or not (Ctrl) for 24 h. Supernatant of LSECs is detected for IL-10, IL-6, IL-12 and IFNγ by Cytometric Bead Array (CBA). **(D)** LSECs from mice hydrodynamically injected with pSM2 plasmid (HBV) or PBS (HDI control) or not (naïve control) are separated at 14dpi and cultured for 24h. Supernatant of LSECs is detected for IL-10, IL-6, IL-12 and IFNγ by Cytometric Bead Array (CBA). Data are representative of 3 independent experiments. Error bars, mean ± SEM. Unpaired t-test and one-way ANOVA are used. **p < 0.01; ***p < 0.001, ns. not significant (p>0.05).

Our previous study has demonstrated that the activation of the toll like receptor 2 (TLR2) signaling pathway in LSECs induced their functional maturation to produce IL12 and triggered CD8 T cell immunity (Liu et al., 2013). Therefore, we examined whether HBeAg induced LSEC activation also by stimulating the TLR2 signaling pathway. LSECs were separated from TLR2 knockout mice, stimulated with rHBeAg and then cocultured with TCR-activated T cells (**Figure 6A**). Our data showed that TLR2 gene ablation in LSECs had no significant influence on the effect of HBeAg-LSEC-mediated T cell immunity, as activated T cells cocultured with HBeAg pretreated TLR2 knockout LSECs still produced significantly increased amounts of IFNγ compared with those cocultured with unstimulated TLR2 knockout LSECs (**Figure 6B**). Besides, LSECs from wild-type mice and TLR2 knockout mice showed no significant difference in triggering CD8 T cell activation (**Figure 6B**). Next, we also examined the production of cytokines, including IL12, IL6, IL10, and IFNγ, by LSECs after HBV or HBeAg exposure. No significant changes in IL12 or IFNγ production were observed in either the HBV-exposed LSECs or HBeAg-LSECs compared with control LSECs (**Figures 6C, D**). In contrast, both HBV- and HBeAg-exposed LSECs produced significantly increased amounts of IL6 and IL10 (**Figures 6C, D**). Taken together, these results demonstrate that HBeAg stimulation induces LSEC to trigger T cell immunity is independent of the TLR2 signaling pathway.

## Discussion

The liver is not only a target of infectious agents, such as hepatotropic viruses, but also a unique immune organ that regulates immunity. However, the mechanisms that determine intrahepatic immune surveillance against infected hepatocytes remain poorly characterized. Liver sinusoidal cells, with their potent immunomodulation functions, serve as sentinel cells to detect microbial infection through pattern recognition receptor activation (Liu et al., 2013) and actively contribute to regulating immune responses for surveillance against pathogens (Wohlleber and Knolle, 2016). LSECs act as a platform for immune cell populations to adhere and are, therefore, in continuous physical contact with all immune cells arrested in the sinusoids. Under physiological conditions, LSECs prevent local T cell activation and the execution of effector functions in physical contact with T cells (Tang et al., 2009; Liu et al., 2013). However, the function of controlling T cell activation of LSECs for hepatic immune regulation may be altered during microbial infection or under inflammatory conditions. Infection with murine cytomegalovirus (MCMV) has been shown to cause functional maturation of LSECs and induce antigen-specific effector CD8 T cells differentiation in the absence of dendritic cells (Kern et al., 2010). Moreover, our previous studies have demonstrated that TLR2 or nucleotide-binding oligomerization domain 1 stimulation transformed tolerogenic LSECs into immunogenic LSECs and activated CD8 T cell response (Liu et al., 2013; Huang et al., 2018a; Huang et al., 2018b). Recently, we report for the first time that intrahepatic HBV exposure could also cause functional maturation of LSECs and abolish their prevention of local T cell activation through HBeAg stimulation (Xie et al., 2021). In the HBV HDI mouse model, the lack of HBeAg presence or TNF-α functional blockade results in compromised intrahepatic HBV-specific CD8 T cell responses and delayed HBV clearance, suggesting that the observed gain in functionality in HBeAg-stimulated LSECs plays an important role in the generation of effective anti-HBV T cell responses and controlling HBV replication in the liver.

In previous studies, extensive screening efforts were made to uncover the costimulatory mechanisms of LSEC-induced T cell activation (Kern et al., 2010; Liu et al., 2013; Bottcher et al., 2014). It has been shown that MCMV-infected LSECs promote antigen-specific effector CD8 T cell differentiation independent of costimulatory molecule CD80/86 (Kern et al., 2010). Instead, LSECs are prone to produce soluble cytokines, such as IL6 (Bottcher et al., 2014) and IL12 (Liu et al., 2013), to promote T cell responses. In our previous study, we have further characterized the characteristics of mature LSECs and identified IL27 and TNF-α as effectors produced by LSECs to facilitate T cell activation (Xie et al., 2021). IL27 is a heterodimeric cytokine of IL6/IL12 that comprises Epstein-Barr virus-induced gene 3 (EBI3) and IL27p28, which signals through a receptor composed of gp130 (utilized by many cytokines, including IL6) and IL27 receptor α (Pflanz et al., 2002; Pflanz et al., 2004). IL27 is mainly produced by APCs, upon infection caused by intracellular pathogens, and can promote innate and adaptive immunity (Yoshida and Hunter, 2015; Povroznik and Robinson, 2020). Consistent with our observation, a previous analysis reported that serum IL27 levels were significantly increased in HBV-infected patients compared with those in healthy individuals, and the level increased further in the presence of HBeAg among the patients (Zhu et al., 2009). TNF-α has also been recognized to play a fundamental role in controlling HBV infection through different mechanisms, including direct inhibition of HBV replication and regulation of anti-HBV immunity in previous studies (Romero and Lavine, 1996; Tzeng et al., 2014; Chyuan et al., 2015). In the study, we blocked the function of TNF-α in HBV exposure mice, and observed that the LSECs of the mice was incompetent to trigger HBV-specific CTLs, thus decelerated the serum HBsAg and HBeAg clearance.

HBeAg is not a structural component of the virus, nor is it required for infectivity or replication, but it is highly conserved in HBV and its related animal viruses (Chang et al., 1987; Chen et al., 1992; Revill et al., 2010). HBeAg has been widely regarded as a key immunomodulator for promoting host innate and adaptive immune tolerance during chronic HBV infection *via* a number of different mechanisms (Kramvis et al., 2018). It has been shown that HBeAg can downregulate TLR expression in hepatocytes and inhibit the TLR signaling pathway in different types of immune cells (Visvanathan et al., 2007; Wu et al., 2009; Lang et al., 2011). It is also believed that HBeAg acts as a T cell tolerogen and regulates the T cell immunity against HBcAg (Chen et al., 2004; Chen et al., 2005), which is important for HBV elimination in humans (Chisari and Ferrari, 1995). HBcAg and HBeAg are indistinguishable in terms of T cell priming and target recognition and, thus, are highly cross-reactive at the CD4 and CD8 T cell responses (Bertoletti et al., 1991; Townsend et al., 1997). Exposure to HBeAg, especially *in utero* or early after birth, elicits neonatal T cell tolerance to both HBeAg and HBcAg and predisposes to viral persistence (Chen et al., 2004; Frelin et al., 2009). Maternal HBeAg could induce an M2-like polarization and upregulation of PD-L1 expression in hepatic macrophages in offspring, which in turn results in an impaired anti-HBcAg CD8 T cell response (Tian et al., 2016). Moreover, it has been shown recently that HBeAg dampens T cell function by inducing the expansion of monocytic myeloid-derived suppressor cells in chronic HBV infection (Yang et al., 2019). However, despite being intensively studied in chronic HBV infection, the immunomodulation function of HBeAg has rarely been examined in the context of acute HBV infection, during which HBeAg is also frequently expressed; however, the infection is efficiently resolved in most infected adults (Ganem and Prince, 2004). A previous *in vivo* study of HBV transgene mice showed that compared to hepatocytes expressing only HBcAg, hepatocytes expressing both HBcAg and HBeAg are more susceptible to HBc/HBeAg-specific CTL-mediated clearance (Frelin et al., 2009), suggesting that the presence of HBeAg facilitates the effector functions of T cells in the liver. Our data in the current study provide direct evidence to demonstrate that HBeAg could also serve as a stimulator to trigger LSEC maturation and prepare the intrahepatic immune microenvironment to facilitate anti-HBV CTL to execute their effector functions.

Taken together, our study emphasizes the importance of HBeAg in exerting LSECs to promote HBV-specific T cell activation in TNF-α signal pathway and gives a new insight into elucidating intrahepatic immune microenvironment regulation during HBV infection.

## Data Availability Statement

The original contributions presented in the study are included in the article/**Supplementary Material**. Further inquiries can be directed to the corresponding author.

## Ethics Statement

The animal study was reviewed and approved by the Experimental Animal Ethics Committee, Tongji Medical College, Huazhong University of Science and Technology.

## Author Contributions

Study concept and design: XX, JZL, DLY, JIL. Acquisition of data: XX, JZL, DZ, WZ. Analysis and interpretation of data: XX, JZL, JIL. Drafting of the article: XX, DY, JIL. Critical revision of the manuscript for important intellectual content: ML, XZ, UD, DY, JIL. Statistical analysis: XX, JIL. Obtained funding: DY, JIL. Administrative, technical, or material support: XY, XF. Study supervision: ML, XZ, UD, DY, JIL. All authors contributed to the article and approved the submitted version.

## Funding

This work is supported by the National Natural Science Foundation of China (81861138044, 82172256, 92169105, 91742114 and 91642118), the National Scientific and Technological Major Project of China (2017ZX10202203), the Integrated Innovative Team for Major Human Diseases Program of Tongji Medical College, HUST, and the Sino-German Virtual Institute for Viral Immunology.

## Conflict of Interest

The authors declare that the research was conducted in the absence of any commercial or financial relationships that could be construed as a potential conflict of interest.

## Publisher’s Note

All claims expressed in this article are solely those of the authors and do not necessarily represent those of their affiliated organizations, or those of the publisher, the editors and the reviewers. Any product that may be evaluated in this article, or claim that may be made by its manufacturer, is not guaranteed or endorsed by the publisher.
