# Supplementary material for: HBeAg Is Indispensable for Inducing Liver Sinusoidal Endothelial Cell Activation by Hepatitis B Virus
Source: Front Cell Infect Microbiol. 2022 Jan 31;12:797915. doi: 10.3389/fcimb.2022.797915 (PMC8842949; doi:10.3389/fcimb.2022.797915)

**HBeAg is indispensable for inducing liver sinusoidal endothelial cell activation by hepatitis B virus**

Xiaohong Xie^1*^, Jinzhuo Luo^1*^, Dan Zhu^1^, Wenqing Zhou^1^, Xuecheng Yang^1^, Xuemei Feng^1^, Mengji Lu^2^, Xin Zheng^1^, Ulf Dittmer^2^, Dongliang Yang^1#^, Jia Liu^1#^

*^1^ Department of Infectious Diseases, Union Hospital, Tongji Medical College, Huazhong University of Science and Technology, Wuhan, Hubei, China*

*^2^ Institute for Virology, University Hospital of Essen, University of Duisburg-Essen, 45122 Essen, Germany*

* These authors contributed equally to this work.

# These authors contributed equally to this work.

**Correspondence to:**

Prof. Dr. Jia Liu, e-mail: [jialiu77@hust.edu.cn](mailto:jialiu77@hotmail.com),

Tel: +8618696159826

Department of Infectious Diseases, Union Hospital, Tongji Medical College, Huazhong University of Science and Technology, Wuhan 430022, China

**Financial support statement:**

This work is supported by the National Natural Science Foundation of China (81861138044, 82172256, 92169105, 91742114 and 91642118), the National Scientific and Technological Major Project of China (2017ZX10202203), the Integrated Innovative Team for Major Human Diseases Program of Tongji Medical College, HUST and the Sino-German Virtual Institute for Viral Immunology.

**Supplementary figure legends**

**Figure S1. Abolishment of LSEC-mediated T cell suppression by hepatitis B virus (HBV) is hepatitis B e antigen (HBeAg)-dependent** C57BL/6 mice are hydrodynamically injected with pBS/HBV1.3 plus pCI-neo/null (HBe^wt^) or pBS/HBV1.3-HBeAg^dep^ plus pCI-neo/null (HBe^del^) or pBS/HBV1.3-HBeAg^dep^ plus pCI-neo/HBeAg (HBe^res^). (A) The HBeAg expression levels of the mice hydrodynamically injected with different combinations of plasmids at different time points. (B) CD8 T cells in the spleen are analyzed for CD69 expression by flow cytometry at 14 dpi. Data are representative of 3 independent experiments. Error bars, mean ± SEM. One-way ANOVA is used. **p<0.01; ns, not significant(p>0.05).

**Figure S2. IL27 blockade in vivo does not result in difference in hepatitis B virus clearance** (A) C57BL/6 mice are hydrodynamically injected with the pSM2 plasmid and intraperitoneally injected with anti-IL27 antibody or isotype control antibody (control). The serum HBsAg and HBeAg levels in the plasmid-injected mice are monitored at indicated time points. Data are shown as the kinetic of HBsAg or HBeAg positive percentage at indicated time points. Four mice were analyzed per group, and at least two independent experiments were performed.

**Figure S3. HBeAg stimulated Kupffer cells (KCs) are incapable of triggering CD8 T cells responses** (A) KCs from naïve mice (n=3) are pretreated with 1 μg/ml or 10 μg/ml rHBeAg or not (Ctrl) for 24 h, and then cocultured with polyclonal stimulated splenocytes at a ratio of 1:2 (KCs to splenocytes). IFNγ production is measured after 48 h. Anti-CD3/anti-CD28–stimulated splenocytes only is used as responder controls (RC). Unstimulated splenocytes is used as negative control (NC). Data are representative of 2 independent experiments. Error bars, mean ± SEM.


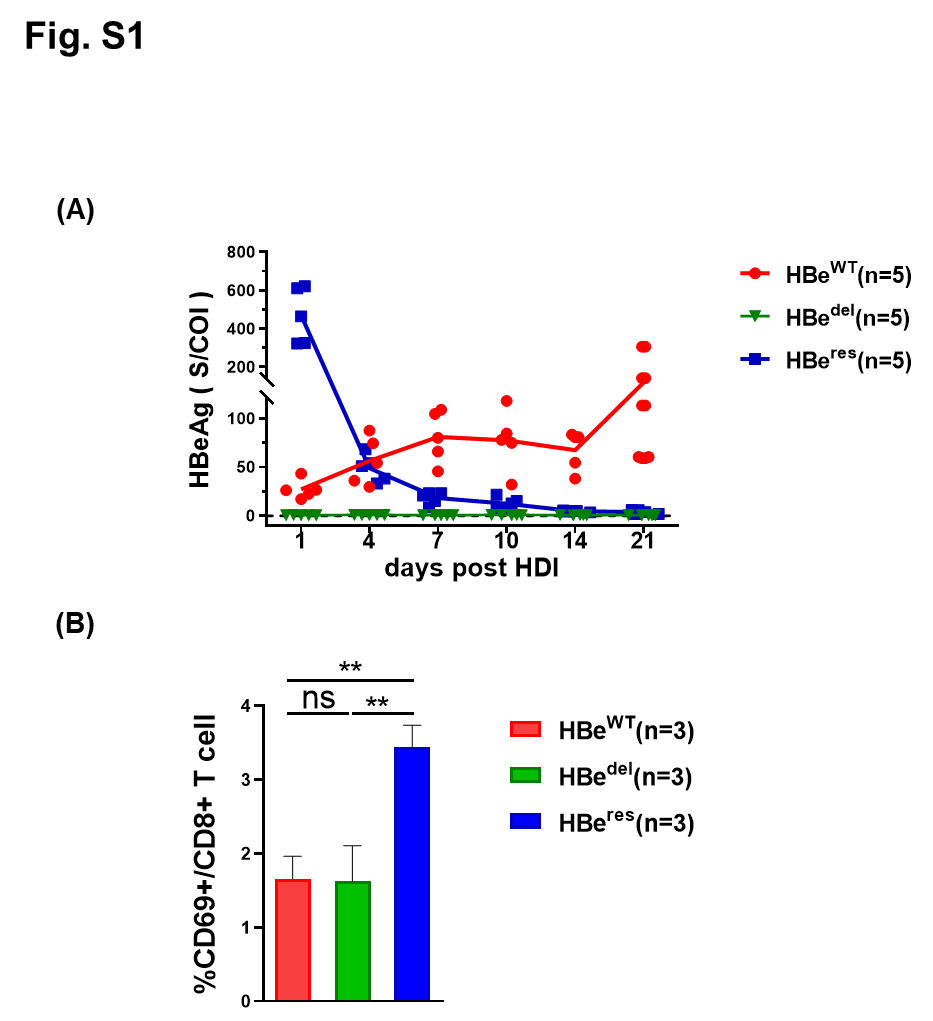


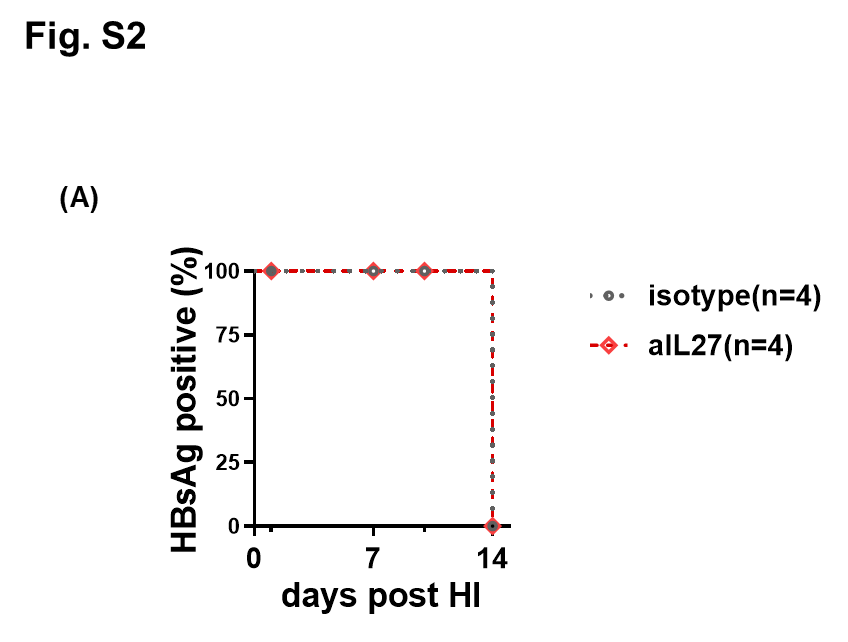


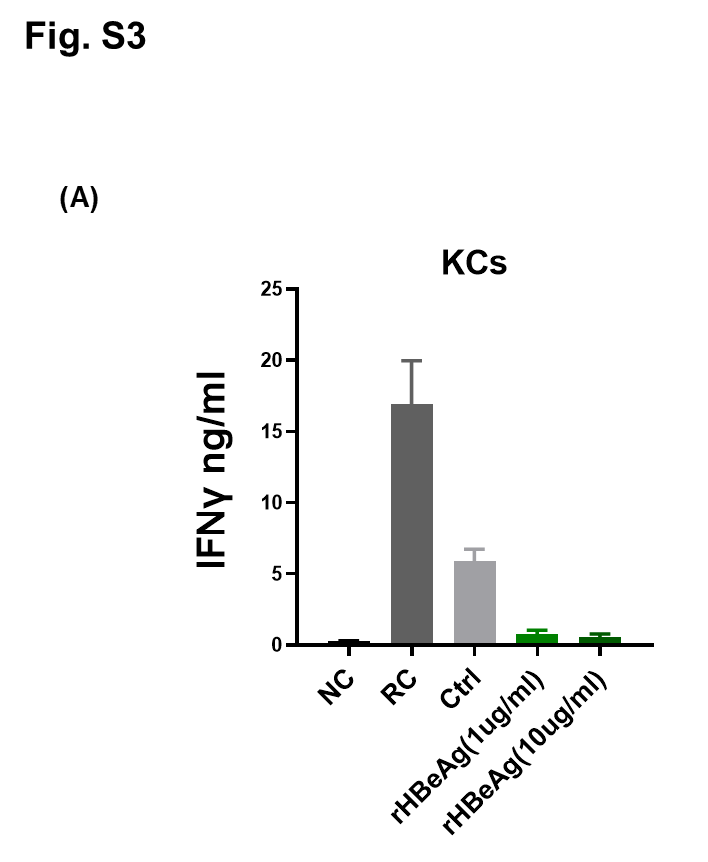


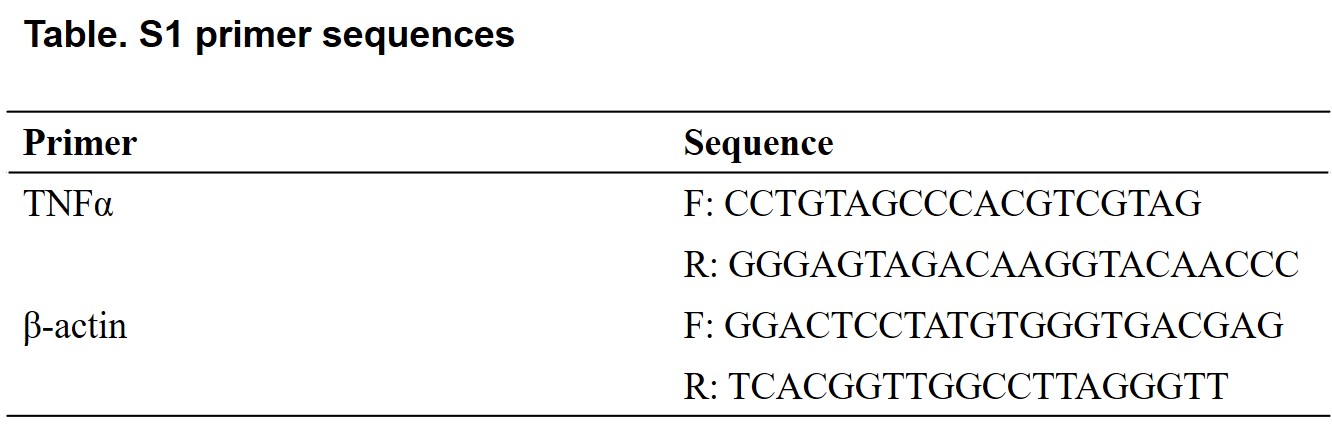


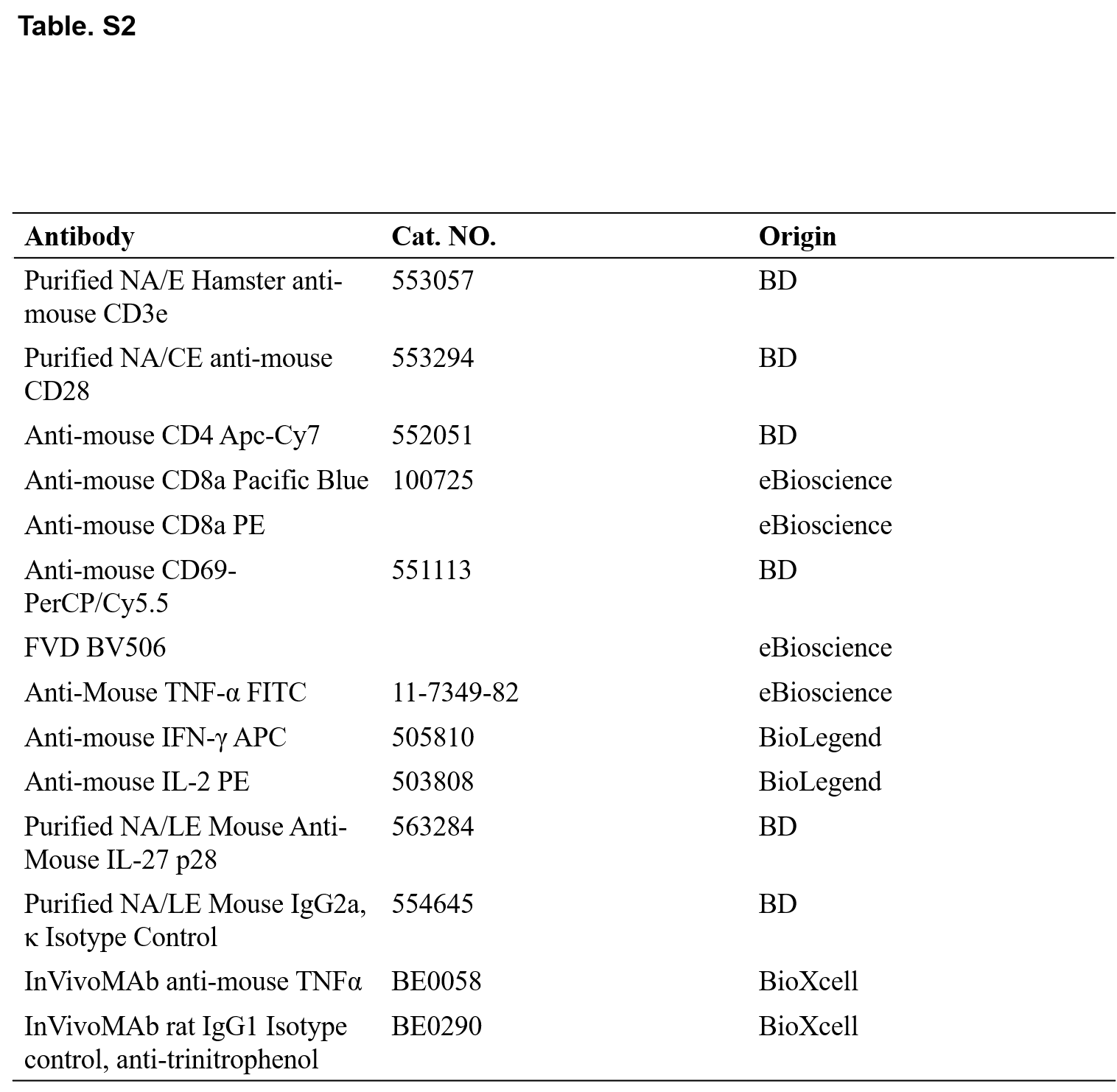

Supplement: Supplementary file 1 [file DataSheet_1.docx]
